# Companion Animal Owners’ Knowledge, Attitudes and Perceptions Regarding Antibiotic Use in Portugal

**DOI:** 10.3390/antibiotics13060533

**Published:** 2024-06-07

**Authors:** Margarida Correia Dias, Russell Alpizar-Jara, Catarina Lavrador, Cátia Marques, Els M. Broens, Elsa L. Duarte

**Affiliations:** 1 Department of Veterinary Medicine, School of Science and Technology, Universidade de Évora, 7004-516 Évora, Portugal; clavrador@uevora.pt (C.L.);; 2 MED–Mediterranean Institute for Agriculture, Environment and Development & CHANGE–Global Change and Sustainability Institute, Universidade de Évora, 7004-516 Évora, Portugal; 3 Department of Mathematics, School of Science and Technology & CIMA Research Centre in Mathematics and Applications, Universidade de Évora, 7004-516 Évora, Portugal; 4 Faculty of Veterinary Medicine, Lusófona University, 376 Campo Grande, 1749-024 Lisbon, Portugal; catia.marques@ulusofona.pt; 5 Associate Laboratory for Animal and Veterinary Sciences (AL4AnimalS), CECAV—Animal and Veterinary Research Centre, University of Trás-os-Montes and Alto Douro, Quinta de Prados, 5000-801 Vila Real, Portugal; 6 Associate Laboratory for Animal and Veterinary Sciences (AL4AnimalS), Centre for Interdisciplinary Research in Animal Health (CIISA), Faculty of Veterinary Medicine, University of Lisbon, 1300-477 Lisbon, Portugal; 7 Genevet, Diagnostic Laboratory and ECVM Satellite Training Center, 1495-191 Carnaxide, Portugal; 8 Department of Biomolecular Health Sciences, Faculty of Veterinary Medicine, Utrecht University, 3584 CS Utrecht, The Netherlands

**Keywords:** antimicrobial resistance, companion animal, owner, antimicrobial stewardship, one health, antimicrobial use

## Abstract

While multiple studies have focused on the motivations surrounding antibiotic prescription among veterinarians, little is known about companion animal owners’ knowledge, attitude and perception (KAP) regarding the topic. A nationwide survey directed toward Portuguese dog and cat owners was conducted online and at veterinary practices to characterize their KAP regarding antibiotics. After database curation, a total of 423 valid submissions were considered. Although 97.9% of respondents stated that they knew what an antibiotic was, 23.5% and 19.2% answered that they were used to treat viral and fungal infections, respectively. Antimicrobial effectiveness was favored over cost when 87.7% of owners agreed they would prefer to spend more money to identify the appropriate antibiotic. Around 87% of respondents recognized antibiotic resistance as a significant health problem and 74.6% strongly agreed/somewhat agreed that antibiotic use in pets may contribute to resistance development. However, only 25.3% recognized that this could promote resistance dissemination, showing little awareness of the interconnection between human and animal health. Moreover, 55.6% of respondents were neutral when asked whether antibiotics used in veterinary medicine were also important for humans. These findings suggest that communication between veterinarians and pet owners can still be improved to further clarify the impact that antibiotic use has in pets from a One-Health perspective, also enabling antimicrobial stewardship interventions.

## 1. Introduction

The discovery and introduction of antimicrobials during the 20th century revolutionized medicine, representing a turning point in human history [1,2,3,4]. Similarly, antimicrobials have become essential tools for the therapy of bacterial diseases in companion animals. 

The rapid emergence of antimicrobial resistance (AMR) over the last 80 years represents a worldwide threat to both humans and animals. The increase in bacterial resistance appears as a side effect of antibiotic exposure due to the selection, development and dissemination of resistant strains [5,6,7,8,9].

Most bacteria and their resistance genes can move within and between humans, animals and the environment. Given this, microbial adaptations to antimicrobial use and other selective pressures within any one sector are reflected in other sectors [10,11,12,13,14].

A unique aspect related to AMR and the risk of resistance transfer associated with companion animals is their close physical contact with humans, which can lead to the transmission of antibiotic resistant microorganisms [15]. Furthermore, improved levels of treatment are devoted to sick animals nowadays, which translates into a significant increase in a pet’s life expectancy and the use of antibiotics. This is of particular concern when considering that the vast majority of antibiotic classes are used in both humans and animals, including critically important antimicrobials (CIAs), with only a few reserved exclusively for humans [11,12,16,17,18,19]. In response to this, comprehensive antimicrobial stewardship programs (ASPs), designed to promote a responsible and judicious use of antibiotics, have been implemented in both human and veterinary medicine. A core element identified in any successful ASP involves the education of prescribing clinicians [20,21,22]. In this context, many studies worldwide have focused on understanding the knowledge, attitude and prescribing drivers among healthcare professionals and medical students, aiming to highlight the main determinants involved in antibiotic misuse [23,24,25,26,27,28,29,30,31]. A similar investigation has been directed toward veterinary students and veterinarians [6,16,32,33,34,35,36,37,38]. 

However, little is known about owners’ knowledge, attitudes and perceptions regarding antibiotic use in companion animals and the dissemination of AMR. Only a few recent studies, from the United Kingdom, Australia and the United States, have focused extensively on pet owners’ motivations and expectations concerning their poorly pet [16,39,40,41,42].

To the best of our knowledge, pet owners’ awareness of antibiotics and AMR in Portugal has never been explored, neither have their attitude or perceptions regarding the administration of antimicrobial drugs to their pets. Overcoming this gap in knowledge may create a considerable opportunity to leverage the trust between pet owners and veterinarians, reducing inappropriate or excessive use of antimicrobial drugs as owners’ expectations or demands could pressure veterinarians to prescribe unnecessary antibiotics. Further to this, acknowledging the differences in responses between demographic groups should support veterinary professionals in tailoring their communication strategy.

## 2. Results

### 2.1. Respondents

After database curation, 423 surveys were validated. Most of the respondents were female (77.5%, 327/423) and 82.5% (348/423) of the surveys were completed by individuals under 55 years old.

Respondents living in urban areas were overrepresented (72.7%, 306/423) when compared to those living in small towns or villages (27.3%, 115/423). A high proportion of respondents (38.7%, 163/423) held a B.Sc. degree and 33.8% (143/423) had obtained a postgraduate educational qualification. Survey respondents who cited a human/animal health background accounted for 42% (177/423).

Less than half of the respondents (42.1%,177/423) were responsible for only one pet. The demographic parameters are detailed in Supplementary Material Table S1.

### 2.2. Pet Owners’ Experience and Expectations

#### 2.2.1. The Most Recent Veterinary Appointment and Expectation of Antibiotic Therapy

Approximately 38.7% (163/421) of the respondents had attended a veterinarian with their pet due to illness within the 4 months preceding completion of the survey. Reflecting upon this visit, most respondents (61.7%, 259/420) stated that they had not expected their pet to need antibiotics to treat its health problem and, curiously, 61.7% (259/420) of the pets consulted had not been prescribed antibiotics on this occasion (Table 1).

The pets of owners who expected their pet to need antibiotics did receive antibiotics more often than the pets of owners who did not expect their pet to need antibiotics (Chi-square test, *p* < 0.001—Supplementary Material Table S2). Only a small minority of the respondents (2.3%, 4/176) felt unhappy with the veterinarian’s decision to provide or withhold antibiotics, while 11.3% (20/176) remained unsure. Culture and susceptibility testing was performed in 41.5% (59/142) of the pets that were prescribed antibiotics, while 46.5% (66/142) of the owners responded that no samples were taken for culture and susceptibility testing.

A significant number of pet owners (44%, 161/366) revealed that, at some point, they had expected antibiotics to be prescribed to their pet and approximately half of these individuals (47.8%, 77/161) had confided this thought to the veterinarian (Supplementary Material Table S3). Overall, most owners appeared to value their veterinarian as a trusted expert, relying on the professional’s clinical judgement and decision to not always prescribe antibiotics to the diseased pet. However, 16.3% (59/361) of the pet owners felt the need for a second opinion after not having antibiotics prescribed. Importantly, this proportion increased up to 23.3% (50/215) when the owner has no health-related professional activity and 34.3% (35/102) when they belonged to the lowest level of education category. The statistical association between the variables “highest education” and “professional activity” with the desire for a second veterinarian opinion was confirmed for a value of *p* < 0.01 (Chi-square test) (Supplementary Material Table S4a,b). 

The majority (82%, 343/418) of the survey respondents stated that they had never given antibiotics to their pet in the absence of an appointment (Supplementary Material Table S5); within the 16.3% (68/418) of respondents who recognized this practice as something they had done before, 66% (45/68) belonged to the highest education category, possessing no human/animal health professional background. Overall, the owners agreed that it is the veterinarians’ responsibility to preserve both human and animal health, revealing little or no difficulty in understanding their professional medical decisions. Nearly 73% (267/366) of the survey respondents would prefer to avoid administering antibiotics to their pets during their life unless strictly necessary (Supplementary Material Table S6). Approximately 80% of the respondents expected the veterinarians to prescribe the treatment that is simultaneously the most convenient and affordable. Despite this, owners continue to favor effectiveness over cost, with the vast majority of respondents (over 85%) revealing they would prefer the veterinarian to select the most suitable antibiotic supported by culture and susceptibility test rather than based on experience (Supplementary Material Table S6).

Even though more than half of the respondents expressed little concern over administering oral treatment to their pets at home, approximately 45% (166/367) stated they would still prefer their pet to receive a single long-acting antibiotic injection. Nevertheless, when comparing the ease and convenience of antibiotic administration versus an increased risk of antimicrobial resistance development, nearly three-quarters of the respondents would prefer not to jeopardize antibiotics’ efficacy (Supplementary Material Table S7).

#### 2.2.2. Antibiotic Use Overlap between Human and Veterinary Medicine

When considering whether to administer antibiotics that are commonly used in human medicine to pets, 53.4% (196/367) of the respondents were in agreement about ensuring their animals receive the treatment they need, even if it implies using human antibiotics (Supplementary Material Table S8). About one-third of respondents did not have an opinion about this subject and the owners with no professional health background appeared overrepresented in this group (75%, 102/136, of the neutral participations belonged to owners with professional activities unrelated with either human or animal health) (Supplementary Material Table S9). Approximately one-third of the respondents remained unsure about using critically important human antibiotics for their pet, while most (62%, 228/367) considered it is the veterinarian’s obligation to put all effort in curing their animal, even though it means using these kinds of antibiotics (Supplementary Material Table S8). Within this population, 55% (127/228) belonged to a professional group with no health background, while 28.5% (65/228) were human health professionals and the remaining worked in animal health (Supplementary Material Table S9).

### 2.3. Pet Owners’ Knowledge of and Opinion on Antibiotic Use

While 98% (375/383) of the respondents stated that they knew what an antibiotic is, 41.7% (160/383) selected at least another microorganism rather than only bacteria as the target for antibiotic treatment (Supplementary Material Table S10).

A total of 61% (236/387) of the pet owners did not think antibiotics are suited to every health problem. Actually, over three-quarters of the survey respondents assigned this preventive role to vaccination protocols, relying on these to reduce the need for antibiotic administration to pets (Supplementary Material Table S11). However, when focusing on antibiotic administration before and following any surgical procedure, 46.6% (179/384) of the owners considered this to be an essential practice, while 22.9% (88/384) had no opinion (Supplementary Material Table S11). Approximately 29% (114/387) of the survey respondents trusted antibiotics to cure any illness, regardless of its nature. Within the respondents who shared this opinion, 41% (46/113) belonged to the lowest education level group, 36% (41/113) had a B.Sc. degree and 23% (26/113) held a higher qualification (Supplementary Material Table S12). Curiously, when comparing the opinions health professionals have on surgical prophylactic antibiotic administration, it becomes clear that human health professionals tended to find it a recommended practice, while veterinarian professionals (DVM and technicians) did not overly rely on antibiotic administration to prevent surgical infections. In summary, 40.7% (64/157) of the professionals with a health background agreed with administering antibiotics following any surgical procedure; however, from these, 82.8% (53/64) worked in the human health field. A total of 50% (115/227) of the pet owners with no professional health background agreed that the administration of antibiotics is justified in any surgical procedure (Supplementary Material Table S13). 

Overall, the growing development of AMR is envisaged by the survey respondents as a meaningful problem to which antibiotic use in companion animal clinical practice may make an important contribution. In this context, 86.4% (332/384) of the respondents related the misuse of antibiotics (inappropriate dosages/length of administration) to the development of resistant strains, while 62.2% (241/387) considered that the use of antibiotics is enough to reduce their effectiveness (presumably due to the emergence of resistance mechanisms), even with adequate prescription and administration (Supplementary Material Table S14).

More than 85% of the pet owners believed that the veterinarian should support their antibiotic choice by culture and antibiogram (Supplementary Material Table S14).

A significant level of unawareness was demonstrated by the pet owners when questioned about the overlap between human and companion animal antibiotic usage, as 31.1% (120/386) of respondents had no opinion regarding the subject (Table 2). Over 56% (217/386) of pet owners acknowledged the shared use of antibiotics in human and veterinary medicine, and 44.4% (170/383) agreed with the fact that antibiotics administered to companion animals are also very important in human medicine.

Upon a more thorough analysis (Table 3), veterinary professionals appeared to possess the clearest opinion in regards to the importance antibiotics administered to companion animals may have in relation to human medicine. The awareness to this fact is also presented in Table 4, bearing in mind the influence of educational background, as more qualified participants were the more informed about this matter.

When focusing on the “One Health” concept, the zoonotic potential of bacteria was corroborated by 69.4% (266/383) of the respondents, while 20.1% (77/383) were not aware that bacteria affecting humans could be transmitted to companion animals and vice versa. Despite this, only a small proportion recognized the negative impact antibiotic administration to pets may have on the people who live with them (31.3%, 121/386) or to humans who do not (25.4%, 97/383).

## 3. Discussion

According to our results, pet owners in Portugal do not want their pets to receive antibiotics unless deemed necessary, trusting their veterinarian to make this judgement. Overall, there is broad awareness of the growing emergence and spread of antimicrobial resistance.

Around 86% of the owners considered the veterinarian’s decision to provide/withhold antibiotics to be the most adequate. Other qualitative studies of pet owners’ knowledge, attitudes and perceptions regarding antimicrobial use have also found a considerable level of recognition and trust in the veterinarian to meet their pets’ medical needs [40,41], which could facilitate a more well-reasoned use of antimicrobials for pets. Along the same line, the respondents with no professional background in health and those integrated in the lower education level were more likely to pursue a second medical opinion before agreeing with the veterinarian’s decision to withhold antimicrobial prescription.

Although the pet owners did not express difficulty in understanding their veterinarian, there does not seem to be such ease in confiding certain thoughts to them, namely the expectation of seeing antibiotics prescribed, as 34.4% recognized they had not shared this expectation with the veterinarian, while 17.5% remained unsure as to whether they had discussed the fact with the clinician. It seems that learning and developing effective communication skills could prove useful in detecting and decoding clients’ particular expectations and perspectives concerning their pet’s treatment, therefore enhancing client satisfaction and compliance of specific recommendations. According to McArthur M. and Fitzgerald, J. [43], increasing the use of open-ended questions during the veterinary appointment could yield valuable information about the client’s thoughts and expectations, improving compliance and narrowing the gap between the veterinarian and the client. Similarly, Stein et al. [39,44] endorsed a solid understanding of pet owners’ preferences as a means to improve patient outcomes.

According to their owners, approximately 46% of the pets received empirical antibiotics prescription without antimicrobial susceptibility testing (AST). Indeed, recent surveys worldwide perceived the expense and delay of AST as barriers to appropriate antibiotic prescription in practice [24,33,38,45,46]. The growing availability of faster and more affordable AST could mitigate this in the near future [47].

Smith et al. [16] pointed out that low levels of knowledge and understanding of antimicrobial resistance amongst pet owners could jeopardize the success of antimicrobial stewardship policies. It seems that health literacy does not overlap completely with higher levels of education: 66% of the respondents who had administered antibiotics to their pets without seeing the veterinarian belonged to the highest education level but did not share a professional health background.

The impact that antibiotic administration to pets may have on people is not well acknowledged among pet owners, disregarding the possible interspecies transmission of resistant bacteria. Only 64% of the respondents considered the veterinarian to bear responsibilities in terms of human health, revealing a lack of understanding about the need for a collaborative approach to fight AMR [48].

Over 85% of the respondents revealed they would prefer the veterinarian to select the most suitable antibiotic supported by microbial culture and AST rather than based on experience. This perception was also reported by Redding and Cole (2019) [40] in the greater Philadelphia area, where initiatives to promote a judicious use of antimicrobials, such as microbial culture and AST, were generally appreciated by pet owners. Similarly, in 2019, Stallwood et al. [49] obtained comparable results after surveying cat owners from the United Kingdom, reporting that 65.8% of the owners would be happy to pay for diagnostic tests to allow the selection of the most appropriate antibiotic. A very approximate proportion of owners in Portugal (39.9%) from those surveyed in Stallwood et al.’s study (38.7%) recalled their pets having any diagnostic test prior to antibiotic prescription. In North America, Stein et al. (2021) [39] suggested further research was necessary in order to evaluate the cost impacts pet owners’ decisions related to antimicrobial selection, since cost was determined as the main driving factor in antimicrobial preference for the majority of dog owners surveyed in their study. 

Even though more than half of the respondents in our study expressed little concern over administering oral treatment to their pets, 45% of the surveyed individuals would still prefer the veterinarian to administer a single-dose long-acting antibiotic to their pet rather than pursuing the oral administration of tablets at home. The reported ease of administration of oral medication by Portuguese pet owners could be related to the higher frequency of dog owners surveyed (48.5%) in comparison to cat owners (25.3%). Stallwood et al. [49], who focused exclusively on surveying cat owners, have certainly found a more unique challenge arising from medicating this species; however, almost 45% of respondents seemed willing to be shown and instructed by their veterinarian on how to best medicate their cat. This could be envisaged by veterinarians as an opportunity to improve compliance and to enable utilization of first-line antibiotics.

When putting into the equation the ease of antibiotic administration and the increased risk of antimicrobial resistance development (in association with the more convenient antibiotics), nearly three-quarters of the owners surveyed prioritized global environmental health, choosing antimicrobial stewardship over convenience. This appears to go against the findings Buckland et al. collected in their 2016 study [50], according to which the antimicrobial usage patterns differed substantially between dogs and cats in the UK. According to the same authors, administration of antibiotics to dogs mainly happened through tablet administration (81% of the population), while 55% cats received their antimicrobial treatment by the parenteral route. On a concerning note, these authors found out cats received proportionally higher usage of third-generation cephalosporins, largely explained by the more frequent use of cefovecin-injectable products (54% of the total cat antibiotic prescriptions versus 1.31% of dog antibiotic prescriptions). A similar study is warranted in Portugal to establish a current baseline for antimicrobial usage, as well as to characterize the frequency, quantity and type of antimicrobials used in cats and dogs.

More than 50% of the surveyed owners agreed with ensuring their animal received the treatment needed, even if it implied using human antimicrobials. Most of the neutral responses (3/4) had their origin in individuals unrelated professionally with either human or animal health. Stein et al. 2021 [39] emphasized that participants with a higher education level were more likely to prefer antibiotics that were “not important” for treating infections in people. Our study did not corroborate this conclusion, as the level of education did not seem to influence this perception to the same extent.

As the prohibition of human antibiotic administration to pets is applied to the most important human antibiotics (an overlap with CIAs), pet owners become less confident in their position and neutral participations increase.

As a rule, Portuguese pet owners believe veterinarians are liable for the treatment of their patients, expecting these professionals to put all effort into curing their pets, even though it might involve prescribing human CIAs. This is in accordance with the findings reported in the context of contemporary international studies and may have a shared reason. As such, according to Smith et al. (2018) [16], for most pet owners, antimicrobial resistance was perceived as a distant and abstract problem of unknown dimensions, which appeared to contrast with the immediacy of their pets’ health. On the other hand, Dickson et al., in 2019 [51], focused on understanding the impact that the relationship owners maintained with their pets had on the emergence and spread of antimicrobial resistance, ascertaining anthropomorphism and emotional attachment to be antecedents of antibiotic decision making.

Further to this, Stein et al. (2021) [39] revealed that the importance of an antimicrobial for treating people was the lowest priority for dog owners in North America, as compared to the cost and ease of administration. Frey et al. (2023) [52] demonstrated that pet owners’ expectations related to antimicrobial use in their pets vary with their knowledge of antimicrobial usefulness and with the recognition of the implications of antimicrobial resistance.

Although 98% of our survey respondents believed that they knew what an antibiotic is, an effort to deepen their comprehension revealed a poor core knowledge on the subject, as 42.7% of the owners selected a microorganism other than bacteria as the target. In fact, Stallwood et al. (2019) [49] obtained a percentage of cat owners as high as 84% who correctly recognized bacteria as the true microorganism upon which antibiotics act. High percentages were also obtained by Scarborough et al. (2021) [41] and Frey et al. in 2023 [52]. On the other hand, Taylor and Walter’s study from 2022 [53], which focused on Colorado pet owners’ perceptions of antimicrobial drug use, reflected comparable poor knowledge on the subject, as approximately 40% of their participants revealed a lack of familiarity with antibiotics’ ineffectiveness against viruses. Although a recent study found that the level of health literacy in Portugal was high [54], this result highlights the need to educate pet owners regarding antimicrobials.

Approximately two-thirds of the respondents recognized that antimicrobials should not be envisaged to prevent diseases. The attribution of this prophylactic purpose to vaccination protocols is an encouraging finding, with over three-quarters the respondents relying on these to reduce the need for antibiotic administration to pets. The above results are in agreement with a Portuguese pilot study published by Prata, J.C. in 2020, where the author found the vaccination rates in the studied pets to be around 90% [55].

Within the population expecting antibiotics to cure any illness, 41% belonged to the lowest level of the education category. A similarly low level of health literacy was also found in the survey by Arriaga et al. [54] within the lowest education categories in Portugal.

There was a higher percentage of health professionals who agreed with antimicrobial usage in the context of surgery among the human health workers. A study about the drivers of the irrational use of antimicrobials in Europe was conducted in 2019 by Machowska and Lundborg [29], making reference to fear as a trigger for irrational prescription. In 2015, Gonzalez et al. corroborated this finding, referring to “fear of the consequences of an eventual infection” as the main attitude affecting both the quantity and the quality of antimicrobial prescription by physicians [56]. It is likely that the same principle could be applied to the surgical field; however, further research into surgical antimicrobial prophylaxis prescribing is warranted in both human and veterinary medicine. Gathering evidence-based data on the usefulness of prophylactic antibiotic administration to pets submitted to surgeries is currently ongoing [57]. As for other EU member states, since January 2022, Portugal has implemented an online platform for the Electronic Prescription in Veterinary Medicine, assuring antimicrobial prescription surveillance within the veterinary field [58,59].

A significant level of unawareness of the overlap between human and companion animal antibiotic usage was observed, as approximately one-third of the survey respondents had no opinion when questioned. Still, the growing development of resistances to antimicrobials is envisaged by most of the survey respondents as a meaningful problem to which antibiotic use in companion animal practice may make an important contribution. We are aware that people who are more concerned about antimicrobial usage and the emergence of resistances are more likely to eventually spend the time answering the survey, thus resulting in a possible sampling bias.

In our study, veterinary professionals appear to possess the clearest opinion about the importance that antimicrobials used within their daily practice play in human medicine. Also, the education level influenced this perception and, as a rule, the more qualified the respondent, the more informed they were on this matter. Redding and Cole (2019) observed that few owners were concerned that the same antimicrobials used for people were also used for pets [40]. Their cross-sectional study related this detachment to the lack of knowledge about the possibility of interspecies transmission of resistant organisms, which had already been inferred by Smith et al. (2018) [16]. Our study raises a similar perspective since approximately 70% of the pet owners corroborated the zoonotic potential of bacteria; however, less than half of them recognized the negative impact antibiotics administered to pets may have on people in or beyond their household. Hence, antimicrobial stewardship messages for the general public should not assume a baseline understanding of the potential interspecies transmission of resistant bacterial strains.

## 4. Material and Methods

A nationwide survey addressed toward pet owners was conducted in Portugal, aiming to create an insight into the owners’ knowledge, attitudes and perceptions around antibiotic use within companion animal practice. Dogs and cats were considered as pets for this purpose. The survey was made available through the online setting (via publications shared on social media websites such as Facebook or through the QR code present on posters made public in veterinary practices’ waiting areas). The survey was open from August 2022 until April 2023.

All the respondents were informed about and agreed to the collection of data for scientific purposes before answering the survey. All the data were processed anonymously. Ethical approval for the study was granted from the ethics committee of the University of Evora (nº 22060 22 June 2023).

The survey, consisting of four groups of questions, considered a range of sociodemographic questions, such as the age group, residential area description, highest education, professional activity and number of pets owned. A special focus was paid to pet owners’ expectations of the veterinary consultation, treatment preferences and trust as well as to the knowledge pet owners demonstrate regarding antibiotic use and the process of development of resistance to these compounds. The survey was composed of closed questions (multiple choice and also a 5-point Likert scale ranging from “strongly disagree” to “strongly agree”).

A total of 42 responses were collected in the form of self-administered paper questionnaires, representing a minority when compared to the total of 677 responses that were retrieved online. Moreover, 226 responses did not fulfil the criterion of “having a pet”, justifying their exclusion. From the remaining 451 responses, and after a more thorough analysis, 28 surveys were disregarded based on having repeated email addresses or appearing unfinished (where over 80% of the survey was left in blank, including the survey core questions represented by groups 3 and 4, related to recent experiences, attitudes and knowledge).

In the survey, and for international readability, the term “antibiotics” was used as a synonym for all the antimicrobial medications directed at bacteria.

Data were recorded in Excel 2021 and analyzed on IBM SPSS version 25, where descriptive analyses and statistical inferences were performed to characterize the disparity in responses and establish a link between sociodemographic variables and owners’ perceptions. The obtained results were analyzed with a Chi-square (X2) test to acknowledge any existing relationship between sociodemographic features and pet owners’ KAP over antimicrobials. The statistical significance threshold was set at a *p*-value ≤ 0.01.

## 5. Conclusions

This survey is, to the best of our knowledge, the first of its kind to provide data on pet owners’ knowledge, attitudes and perceptions around antimicrobial usage in Portugal within companion animal practice.

In small animal clinical practice, an active effort has been applied to reduce the use of antimicrobials; however, there is still space for a further decrease in prescribing practices. Whether they self-administer antibiotics or request veterinarians to prescribe antibiotics for their animal, pet owners show a lack of knowledge that antibiotic resistance can spread among pets, humans and the environment. Based on the high level of trust in veterinarians reported by the surveyed participants, there seems to exist a good opportunity for veterinary surgeons to encourage pet owners to pursue increased diagnostics through improved communication, reducing and refining the use of antimicrobials. Communication strategies regarding the use of antimicrobials in companion animal practice must be tailored in order to meet owners’ preferences and expectations. Acknowledging the behavioral dynamics behind pet owners’ expectations of veterinary treatment should yield additional tools in this regard. Additionally, electronic prescribing, fast and inexpensive AST, and promoting the usage of consensual prescription guidelines could further enhance antibiotic stewardship.

Although the present study did not focus on identifying the motivations among pet owners, it should certainly be valued as a cornerstone of the successful implementation of any antimicrobial stewardship program within companion animal practice.

## Figures and Tables

**Table 1:** Recent veterinarian appointment—Expectations of therapy and satisfaction with the treatment decision.

		n	Frequency
When did you last take your pet to the vet for an illness? (Missing 0.5%, N = 421)	Less than 4 months ago	163	38.7%
	4–12 months ago	96	22.8%
	Over 12 months ago	115	27.3%
	My pet has never been to the vet for an illness	47	11.2%
Before being prescribed any medication, did you expect your pet to need antibiotics for its problem? (Missing 0.7%, N = 420)	Yes	88	21.0%
	No	259	61.7%
	Unsure	73	17.3%
Did your pet receive antibiotics (Missing 0.7%, N = 420)	Yes	142	33.8%
	No	259	61.7%
	Unsure	19	17.3%
Did you feel the decision to prescribe/not prescribe antibiotics to your pet was the most adequate? (Missing 58.4%, N = 176)	Yes	152	86.4%
	No	4	2.3%
	Unsure	20	11.4%
In case antibiotics were prescribed to your pet, did the veterinarian collect any sample to check which antibiotic would be the most suitable? (Missing 0%, N = 142)	Yes	59	41.5%
	No	66	46.5%
	Unsure	17	12.0%

**Table 2:** Pet owners’ knowledge about the impact of antibiotic use in veterinary medicine of companion animals on humans.

	Strongly Agree	Somewhat Agree	Neutral	Somewhat Disagree	Strongly Disagree
The antibiotics used in the treatment of companion animals are not used in human medicine (Missing 8.7%, N = 386)	3.9% (15/386)	8.8% (34/386)	31.1% (120/386)	17.1% (66/386)	39.1% (151/386)
The administration of antibiotics to your pet may have a negative impact to your own health (Missing 8.7%, N = 386)	9.8% (38/386)	21.5% (83/386)	25.1% (97/386)	11.9% (46/386)	31.6% (122/386)
The administration of antibiotics to your pet may have a negative impact to people who do not cohabitate with you (Missing 9.5%, N = 383)	7.6% (29/383)	17.8% (68/383)	23.4% (90/383)	12% (46/383)	39.2% (150/383)
The bacteria which affect companion animals may be transmitted to humans and vice-versa (Missing 9.5%, N = 383)	32.6% (125/383)	36.8% (141/383)	20.1% (77/383)	6.8% (26/383)	3.7% (14/383)
The most frequently used antibiotics in veterinary medicine are also very important in human medicine (Missing 9.5%, N = 383)	20.6% (79/383)	23.8% (91/383)	46.5% (178/383)	6.8% (26/383)	2.3% (9/383)

**Table 3:** Pet owners’ awareness of the antibiotic usage overlap between veterinary and human medicine—Differences by professional activity category (human health background, veterinary health background and no health background).

Professional Activity	The Most Frequently Used Antibiotics in Veterinary Medicine Are Also Very Important in Human Medicine					
	Strongly Agree	Somewhat Agree	Neutral	Somewhat Disagree	Strongly Disagree	Total
Human health	7.6% (29/383)	6.3% (24/383)	1.6% (6/383)	0.26% (1/383)	9.1% (35/383)	24.8% (95/383)
Animal health	6.0% (23/383)	5.5% (21/383)	1.6% (6/383)	0.5% (2/383)	2.6% (10/383)	16.2% (62/383)
Other	7.0% (27/383)	12.0% (46/383)	3.7% (14/383)	1.6% (6/383)	34.7% (133/383)	59.0% (226/383)
Total	23.8% (91/383)	20.6% (79/383)	6.8% (26/383)	2.3% (9/383)	46.5% (178/383)	100% (N = 383)

**Table 4:** Pet owners’ awareness of the antibiotic usage overlap between veterinary and human medicine—Differences by education level.

Highest Education	The Most Frequently Used Antibiotics in Veterinary Medicine Are Also Very Important in Human Medicine					
	Strongly Agree	Somewhat Agree	Neutral	Somewhat Disagree	Strongly Disagree	Total
Elementary education, high school, technological specialization course	2.9% (11/383)	5.7% (22/383)	2.6% (10/383)	0.8% (3/383)	15.4% (59/383)	27.4% (105/383)
B.Sc. Degree	8.6% (33/383)	7.8% (30/383)	2.9% (11/383)	1.0% (4/383)	19.6% (75/383)	39.9% (153/383)
Post-graduate qualification, M.Sc. Degree, Ph.D.	9.1% (35/383)	10.2% (39/383)	1.3% (5/383)	0.5% (2/383)	11.5% (44/383)	32.6% (125/383)
Total	20.6% (79/383)	23.8% (91/383)	6.8% (26/383)	2.3% (9/383)	46.5% (178/383)	100% (N = 383)

## Data Availability

Research data are available within text and Supplementary Material.

## Supplementary Materials

The following supporting information can be downloaded at https://www.mdpi.com/article/10.3390/antibiotics13060533/s1.
