# Supplementary material for: Companion Animal Owners’ Knowledge, Attitudes and Perceptions Regarding Antibiotic Use in Portugal
Source: Antibiotics (Basel). 2024 Jun 7;13(6):533. doi: 10.3390/antibiotics13060533 (PMC11201214; doi:10.3390/antibiotics13060533)
Supplement: Supplementary file 1 [file antibiotics-13-00533-s001.zip › antibiotics-3024852-supplementary.pdf]

Table S1. Demographic parameters of the 423 included survey participants.

**Category**		**n**	**Frequency**
Gender (Missing 0.2%, N=422)	Female	327	77.5%
	Male	95	22.5%
Age group (Missing 0.2%, N=422)	35 or younger	168	39.8%
	35-55	180	42.7%
	55 or older	74	17.5%
Residential area description (Missing 0.5%, N=421)	City and suburbs	306	72.7%
	Small town	52	12.3%
	Village	63	15.0%
Highest education (Missing 0.5%, N=421)	Elementary education, high school or technological specialisation course	115	27.3%
	BSc degree	163	38.7%
	Post-graduate qualification: MSc, PhD	143	34.0%
Field of work (Missing 0.5%, N=421)	Human health	104	24.7%
	Animal health	73	17.3%
	Other	244	58.0%
Number of pets owned (Missing 0.7%, N=420)	1	177	42.1%
	2	99	23.6%
	3 or more	144	34.3%
Pets owned	Cat	107	25.3%
	Dog	205	48.5%
	Cat and dog	111	26.2%

Table S2. Expectation and prescription of antibiotics during the most recent veterinary appointment due to animal illness.

		Did your pet receive antibiotics?		
		No	Yes	Total
**Before being prescribed any medication, did you expect your pet to need antibiotics?**	No	63.4% (213/336)	11.3% (38/336)	74.7% (251/336)
	Yes	2.7% (9/336)	22.6% (76/336)	25.3% (85/336)
	Total	66.1% (222/336)	33.9% (114/336)	100% N=336

Table S3. Pet owners' expectations regarding antibiotic prescription by the veterinarian.

	**Yes**	**No**	**Unsure**
Have you ever felt your pet needed antibiotics at a particular moment? (Missing 13.5%, N=366)	44% (161/366)	42.1% (154/366)	13.9% (51/366)
Did you share this thought with the veterinarian? (Missing 0%, N=161)	47.8% (77/161)	34.2% (55/161)	17.4% (28/161)
Have you ever felt disappointed with the veterinarian's decision of not prescribing antibiotics to your pet? (Missing 13.9%, N=364)	6.3% (23/364)	93.7% (341/364)	0% (0/364)
If the veterinarian prescribes no antibiotics to your poorly pet, do you believe you may need to pursue a second opinion? (Missing 14.7%, N=361)	16.3% (59/361)	83.7% (302/361)	0% (0/361)

Table S4a and S4b. Crosstabulation of variable "professional activity/highest education" and variable "pet owners' reaction towards the veterinarian's decision to withhold antibiotics".

Table S4a.

**Professional activity**	**If the veterinarian prescribes no antibiotic to your poorly pet, do you believe you need to pursue a second opinion?**		
	No	Yes	Total
Human health	22.2% (80/361)	1.9% (7/361)	24.1% (87/361)
Animal health	15.8% (57/361)	0.5% (2/361)	16.3% (59/361)
Other	45.7% (165/361)	13.9% (50/361)	59.6% (215/361)
Total	83.7% (302/361)	16.3% (59/361)	100% N=361

Table S4b.

**Highest education**	**If the veterinarian prescribes no antibiotic to your poorly pet, do you believe you need to pursue a second opinion?**		
	No	Yes	Total
Elementary education, high school, technological specialisation course	18.6% (67/360)	9.7% (35/360)	28.3% (102/360)
BSc degree	35.3% (127/360)	4.4% (16/360)	39.7% (143/361)
Post-graduate qualification, MSc degree, PhD	29.7% (107/360)	2.2% (8/360)	31.9% (115/360)
Total	83.7% (301/360)	16.3% (59/360)	100% N=360

Table S5. Pet owners' attitudes regarding antibiotic administration to their pets without veterinary appointment/advice.

	Yes	No	Unsure
Have you ever administered antibiotics to your pet based on the veterinarian's previous advice but without a face-to-face appointment? (Missing 1.2%, N=418)	16.3% (68/418)	82.0% (343/418)	1.7% (7/418)
Have you ever administered antibiotics to your pet without the veterinarian's advice? (Missing 0.7%, N=420)	5.0% (21/420)	94.5% (397/420)	0.5% (2/420)

Table S6. Pet owners' expectations regarding the veterinarian's responsibilities towards their pets' treatment.

	Strongly agree	Somewhat agree	Neutral	Somewhat disagree	Strongly disagree
I often struggle to understand my veterinarian's explanations (Missing 13.2%, N=367)	2.2% (8/367)	8.7% (32/367)	9.0% (33/367)	21.0% (77/367)	59.1% (217/367)
The veterinarian must look after animal's health (Missing 8.5%, N=387)	92.8% (359/387)	5.2% (20/387)	2.0% (8/387)	0% (0/387)	0% (0/387)
The veterinarian must look after human's health (Missing 8.5%, N=387)	64.3% (249/387)	18.1% (70/387)	7.5% (29/387)	2.8% (11%387)	7.2% (28/387)
It is the veterinarian's obligation to ensure the treatment is as convenient as possible to the pet owner (Missing 13.5%, N=366)	46.7% (171/366)	34.2% (125/366)	6.0% (13/366)	8.2% (30/366)	7.4% (27/366)
It is the veterinarian's obligation to ensure the treatment is as affordable as possible to the pet owner (Missing 13.5%, N=366)	35.2% (129/366)	44.0% (161/366)	3.6% (22/366)	10.9% (40/366)	3.8% (14/366)
Even if it means to spend more money, I would prefer to perform a sensitivity test in order to select the best antibiotic for my pet's illness (Missing 13.2%, N=367)	53.7% (197/367)	31.9% (117/367)	6.3% (23/367)	6.5% (24/367)	1.6% (6/367)
I'd rather my pet didn't receive antibiotics unless strictly necessary (Missing 13.5%, N=366)	41.8% (153/366)	31.1% (114/366)	16.4% (60/366)	7.9% (29/366)	2.7% (10/366)

Table S7. Owners' perceptions and preferences regarding their own role in the administration of antibiotics to their pets.

	Strongly agree	Somewhat agree	Neutral	Somewhat disagree	Strongly disagree
Having to administer tablets to my pet is extremely difficult (Missing 13.2%, N=367)	12.5% (46/367)	26.7% (98/367)	4.9% (18/367)	22.9% (84/367)	33.0% (121/367)
I'd prefer my pet to receive a long-acting injectable antibiotic in a single dose, than being prescribed tablets to administer at home (Missing 13.2%, N=367)	19.1% (70/367)	26.2% (96/367)	32.7% (120/367)	15.5% (57/367)	6.5% (24/367)
If my pet appears to be cured, I stop the antibiotic administration before the end of the prescribed course (Missing 13.2%, N=367)	0.8% (3/367)	1.6% (6/367)	2.7% (10/367)	6.8% (25/367)	88.0% (323/367)
I'd prefer the veterinarian to prescribe an easier to give antibiotic (e.g. lower frequency of administration, better palatability) even if it raises a higher risk of developing environmental resistances (Missing 13.2%, N=367)	3.8% (14/367)	10.4% (38/367)	12.8% (47/367)	22.1% (81/367)	51.0% (187/367)

Table S8. Pet owners' opinion regarding the use of antibiotics used in humans in the treatment of companion animals.

	Strongly agree	Somewhat agree	Neutral	Somewhat disagree	Strongly disagree
Antibiotics used in human medicine should be forbidden in the treatment of companion animals (Missing 13.2%, N=367)	2.7% (10/367)	6.8% (25/367)	37.1% (136/367)	16.3% (60/367)	37.1% (136/367)
Only the antibiotics used to treat serious human hospital infections should be forbidden in the veterinary treatment of companion animals (Missing 13.5%, N=366)	3.3% (12/366)	11.2% (41/366)	47.0% (172/366)	11.7% (43/366)	26.8% (98/366)
It is the veterinarian's obligation to treat my poorly pet, even though it may involve prescribing antibiotics which are normally saved for critical cases in humans (hospital use only antibiotics) (Missing 13.2%, N=367)	33.8% (124/367)	28.3% (104/367)	26.2% (96/367)	6.3% (23/367)	5.4% (20/367)

Table S9. Pet owners' expectation regarding the use of critical human antibiotics in the treatment of companion animals – differences by professional activity category (health background versus non health background).

	It is the veterinarian's obligation to treat my poorly pet, even though it may involve prescribing antibiotics which are normally saved for critical cases in humans (hospital use only antibiotics)					
Professional activity	Strongly agree	Somewhat agree	Neutral	Somewhat disagree	Strongly disagree	Total
Human health	8.7% (32/367)	9.0% (33/367)	3.3% (12/367)	1.6% (6/367)	1.4% (5/367)	24.0% (88/367)
Animal health	4.6% (17/367)	5.2% (19/367)	2.5% (9/367)	2.2% (8/367)	1.9% (7/367)	16.3% (60/367)
Other	20.4% (75/367)	14.2% (52/367)	20.4% (75/367)	2.5% (9/367)	2.2% (8/367)	59.7% (219/367)
Total	33.8% (124/367)	28.3% (104/367)	26.2% (96/367)	6.3% (23/367)	5.4% (20/367)	100% (N=367)

Table S10. Pet owners' knowledge about antibiotic use and microbiology.

		n	Frequency
**Antibiotics are used to treat** (Missing 0%, N=423)	Viral infections	52	13.9%
	Fungal infections	38	10.1%
	Bacterial infections	215	57.3%
	Fungal and bacterial infections	34	9.1%
	Viral and bacterial infections	36	9.6%

Table S11. Pet owners' knowledge about the antibiotic role and its potential as a preventative measure in companion animal clinical practice.

	Strongly agree	Somewhat agree	Neutral	Somewhat disagree	Strongly disagree
More often than not, antibiotics contribute to the cure of the pet regardless of the diagnosis (Missing 8.5%, N=387)	7.0% (27/387)	22.5% (87/387)	9.6% (37/387)	17.6% (68/387)	43.4% (168/387)
The antibiotics should be used to prevent diseases (Missing 8.7%, N=386)	10.1% (39/386)	12.4% (48/386)	9.3% (36/386)	14.5% (56/386)	53.6% (207/386)
Antibiotic administration before and after any surgical procedure is essential (even for the straightforward and quick surgeries, e.g. pet neutering) (Missing 9.2%, N=384)	19.0% (73/384)	27.6% (106/384)	22.9% (88/384)	14.3% (55/384)	16.1% (62/384)
The existence of vaccination protocols for pets may reduce the need for antibiotic use in veterinary medicine (Missing 9.2%, N=384)	52.1% (200/384)	26.0% (100/384)	12.8% 49/384)	4.2% (16/384)	4.9% (19/384)

Table S12. Pet owners' expectation regarding the contribution antibiotics represent to the cure of the pet regardless of the diagnosis – differences by level of education.

	**More often than not, antibiotics contribute to the cure of the pet regardless of the diagnosis**					
**Highest education**	**Strongly agree**	**Somewhat agree**	**Neutral**	**Somewhat disagree**	**Strongly disagree**	**Total**
Elementary education, high school or technological specialisation course	4.1% (16/386)	7.8% (30/386)	4.7% (18/386)	5.2% (20/386)	5.7% (22/386)	27.5% (106/386)
BSc Degree	1.8% (7/386)	8.8% (34/386)	2.8% (11/386)	7.5% (29/386)	18.7% (72/386)	39.6% (153/386)
Post-graduate qualification, MSc Degree, PhD	0.8% (3/386)	6.0% (23/386)	2.1% (8/386)	4.9% (19/386)	19.2% (74/386)	32.9% (127/386)
Total	22.5% (87/386)	6.7% (26/386)	9.6% (37/386)	17.6% (68/386)	43.5% (168/386)	100% (N=386)

Table S13. Pet owners' view regarding the use of antibiotics within the surgical context – differences by professional activity category (human health background, veterinary health background and no health background).

	**Antibiotic administration before and after any surgical procedure is essential (even for the straightforward and quick surgeries, e.g. pet neutering)**					
**Professional activity**	**Strongly agree**	**Somewhat agree**	**Neutral**	**Somewhat disagree**	**Strongly disagree**	**Total**
Human health	3.7% (14/384)	10.2% (39/384)	3.4% (13/384)	4.4% (17/384)	3.1% (12/384)	24.7% (95/384)
Animal health	0.26% (1/384)	2.6% (10/384)	0.8% (3/384)	5.5% (21/384)	7.0% (27/384)	16.1% (62/384)
Other	15.1% (58/384)	14.8% (57/384)	18.8% (72/384)	4.4% (17/384)	6.0% (23/384)	59.1% (227/384)
Total	19% (73/384)	27.6% (106/384)	22.9% (88/384)	14.3% (55/384)	16.1% (62/384)	100% (N=384)

Table S14. Pet owners' knowledge about antibiotic resistance development.

	**Strongly agree**	**Somewhat agree**	**Neutral**	**Somewhat disagree**	**Strongly disagree**
The development of antibiotic resistances is not a significant problem, therefore I don't need to worry about it (Missing 8.5%, N=387)	0.8% (3/387)	3.6% (14/387)	7.2% (28/387)	7.5% (29/387)	80.9% (313/387)
The use of antibiotics in companion animals may contribute to the development of resistant microorganisms (Missing 8.5 %, N=387)	51.2% (198/387)	24.8% (96/387)	19.4% (75/387)	2.8% (11/387)	1.8% (7/387)
The prescription of antibiotics may contribute to reduce their effectiveness (Missing 8.5%, N=387)	28.9% (112/387)	33.3% (129/387)	14.2% (55/387)	15.2% (59/387)	8.3% (32/387)
The prescription of any antibiotic should be preceded by a test which proves the antibiotic is the most suitable for the pet's illness (Missing 8.5 %, N=387)	54.0% (209/387)	31.8% (123/387)	8.5% (33/387)	5.4% (21/387)	0.3% (1/387)
The administration of inappropriate dosages or an insufficient length of treatment with antibiotic may contribute to develop microorganisms which are resistant to these antibiotics (Missing 9.2 %, N=384)	70.3% (270/384)	16.1% (62/384)	10.7% (41/384)	2.1% (8/384)	0.8% (3/384)
